# ZEB1 Defines the Interaction Between Breast Cancer Cells and Macrophages by Upregulating the Expression of CCL2 Cytokine

**DOI:** 10.3390/ijms27209194

**Published:** 2026-10-16

**Authors:** Oleg Semenov, Ivan Nevzorov, Sergei Silonov, Alexandra Daks, Aliya Kabasheva, Alexander Nazarov, Daria Kriger, Ivan Kuneev, Alexey Petukhov, Yulia Gnennaya, Eugene Tulchinsky, Oleg Shuvalov, Nickolai Barlev

**Affiliations:** 1 Institute of Cytology of the Russian Academy of Sciences, St. Petersburg 194064, Russia; semyonov.somspb@yandex.ru (O.S.); ban140598@gmail.com (I.N.); silonovsa25@yandex.ru (S.S.); alexandra.daks@gmail.com (A.D.); anazarov2021@gmail.com (A.N.); oleg8988@mail.ru (O.S.); dkriger@incras.ru (D.K.); gnennaya1996@mail.ru (Y.G.); 2 Department of Biomedical sciences, School of Medicine, Nazarbayev University, Astana 010000, Kazakhstan; aliya.kabasheva@nu.edu.kz (A.K.); eugene.tulchinsky@nu.edu.kz (E.T.); 3 National Laboratory of Astana, Astana 010000, Kazakhstan

**Keywords:** EMT, ZEB1, CCL2, tumor microenvironment, breast cancer, macrophages

## Abstract

The survival of disseminating tumor cells at the invasive edge and in metastatic foci depends on their ability to counteract anti-tumor immunity. The epithelial–mesenchymal transition (EMT) process is associated with the recruitment of immune-suppressive myeloid cells. The onset of the EMT gene expression program is controlled by several transcriptional master-regulators, including ZEB1. Using engineered breast cancer cell lines with different statuses of ZEB1, we examined the effects of ZEB1 on the expression of different pro-inflammatory cytokines. Our results suggest that ZEB1 plays an important role in regulating the expression of CCL2 (C-C motif ligand 2, a.k.a. MCP-1), the main chemoattractant of monocytes. Forced expression of ZEB1 in epithelial MCF-7 cells significantly increased the expression level of CCL2, which was further enhanced by TNF-α. On the contrary, knock-out of *ZEB1* in pseudo-mesenchymal MDA-MB-231 cells led to a complete loss of CCL2 expression. Moreover, this effect was not restored by TNF-α induction. Also, positive ZEB1 status in breast cancer cells promoted the chemoattraction of THP-1 macrophages in co-culture experiments. Analysis of publicly available ChIP-seq data implicated ZEB1 in the regulation of *CCL2* gene transcription. Taken together, these data suggest that ZEB1 is involved in the formation of the EMT-associated pro-tumor immune microenvironment.

## 1. Introduction

The transcription factor ZEB1 (Zinc finger E-box binding protein) is one of the most well-studied regulators of the epithelial–mesenchymal transition (EMT) [1,2,3,4]. This process is pivotal for the development of metastases, providing the opportunity for individual clones to leave the primary tumor and disseminate throughout the body, forming the basis for future metastases [1,5]. Following the dissemination, EMT cells undergo the reverse process, mesenchymal to epithelial transition (MET), to form secondary tumors in distant organs [1,5,6]. In general, carcinoma cells exhibit so-called mixed epithelial–mesenchymal phenotypes which may be characterized by different gene expression profiles [1,5]. Thus, EMT can be considered part of the cell epithelial–mesenchymal plasticity, regulated by the activity of specific transcription factors, including ZEB1 [1,5]. The ability of ZEB1 to bind DNA is mediated by two C2H2-type flanking zinc finger clusters that specifically recognize E-box-like promoter elements. ZEB1 is able to act as both an activator and a repressor of transcription, recruiting chromatin modifiers through interaction domains, such as Smad-(SID), CtBP-(CID), and p300-P/CAF (CBD) interaction domains [3,7,8]. Accordingly, ZEB1 can directly suppress the expression of epithelial genes such as E-cadherin [9,10] and activate transcription of mesenchymal genes [11]. In addition, ZEB1 can indirectly regulate a large number of processes involved in the development of malignant tumors such as stemness [12,13], resistance to cytotoxic therapy [3,14,15], autophagy [16], and others. There is data showing the important role of ZEB1 in the development of breast cancer [17], which is the most common cause of cancer-associated mortality in women in developed countries [18].

One of the main conditions for the development of cancerous tumors is the ability of malignant cells to evade the antitumor immune response [19]. Accordingly, various mechanisms are utilized by cancer cells to suppress cytotoxic immunity. For example, malignant transformation can lead to the exposure of molecules that inhibit the activity of CD8^+^ cytotoxic T lymphocytes, primarily PD-L1, on the outer cell membrane. For such cases, checkpoint immunotherapy with inhibitors of PD-L1 showed very promising results [20].

On the other hand, the tumor microenvironment is able to suppress the initial cytotoxic immune response, converting it into chronic inflammation. In that case, active infiltration by the immune cells of various origins may occur. The variety of leucocytes often includes macrophages, neutrophils, myeloid-suppressor cells, type 2 T-helper cells, etc., and their relatively high numbers can be associated with unfavorable clinical prognosis [21]. A widespread mechanism for the formation of a pro-tumorigenic microenvironment is the recruitment of blood monocytes by cancer cells using the chemokine CCL2 [22,23]. Monocytes differentiate into macrophages and acquire the alternatively activated M2 phenotype, promoting further tumor development [24]. Macrophages secrete a cytokine profile that reduces CD8^+^ T cell activity and supports cancer cell survival and drug resistance [24]. In this respect, one of the major pro-inflammatory cytokines produced by M1-macrophages, TNF-α, was shown to play a pro-tumorigenic role [25]. The published data demonstrates its ability to promote breast cancer cell stemness [26], drug resistance and metastatic capacity [27]. Cancer cells, in turn, are able to increase CCL2 expression in response to pro-inflammatory cytokines, particularly TNF-α [28,29,30]. So, a tumor-promoting positive feedback loop including mutual TNF-α and CCL2 upregulation was shown for breast cancer [31]. In this regard, the role of CCL2 in maintaining a population of pro-tumorigenic macrophages is widely studied [23,32,33].

It should be noted that there is evidence showing that transcription factors associated with EMT can influence the expression of cytokine coding genes, including *CCL2*, thereby regulating the tumor microenvironment [34,35,36]. Consistent with this notion, ZEB1 was shown to regulate the expression of IL6 and IL8 cytokines in breast cancer cells [37].

To gain further insight into the role of ZEB1 in regulating cancer immunity, we performed an unbiased screening of cytokines and chemokines secreted by the luminal A breast cancer cell line MCF-7 following inducible expression of ZEB1. We identified *CCL2* as a potential transcriptional target of ZEB1 in both MCF-7 cells with inducible ZEB1 expression and in the triple-negative, mesenchymal-like breast cancer cell line MDA-MB-231, which expresses ZEB1 endogenously. We further examined the reciprocal interactions between THP-1-derived macrophages and breast cancer cells with opposing ZEB1 expression statuses under co-culture conditions. Finally, we investigated the potential involvement of ZEB1 in the transcriptional regulation of the *CCL2* gene. Collectively, our findings support a model in which ZEB1 promotes the recruitment of cancer-associated macrophages through CCL2 secretion, thereby contributing to the establishment and maintenance of the EMT phenotype.

## 2. Results

### 2.1. ZEB1 Expression Induction Leads to CCL2 Upregulation in MCF-7 Cells

**Figure 1:**
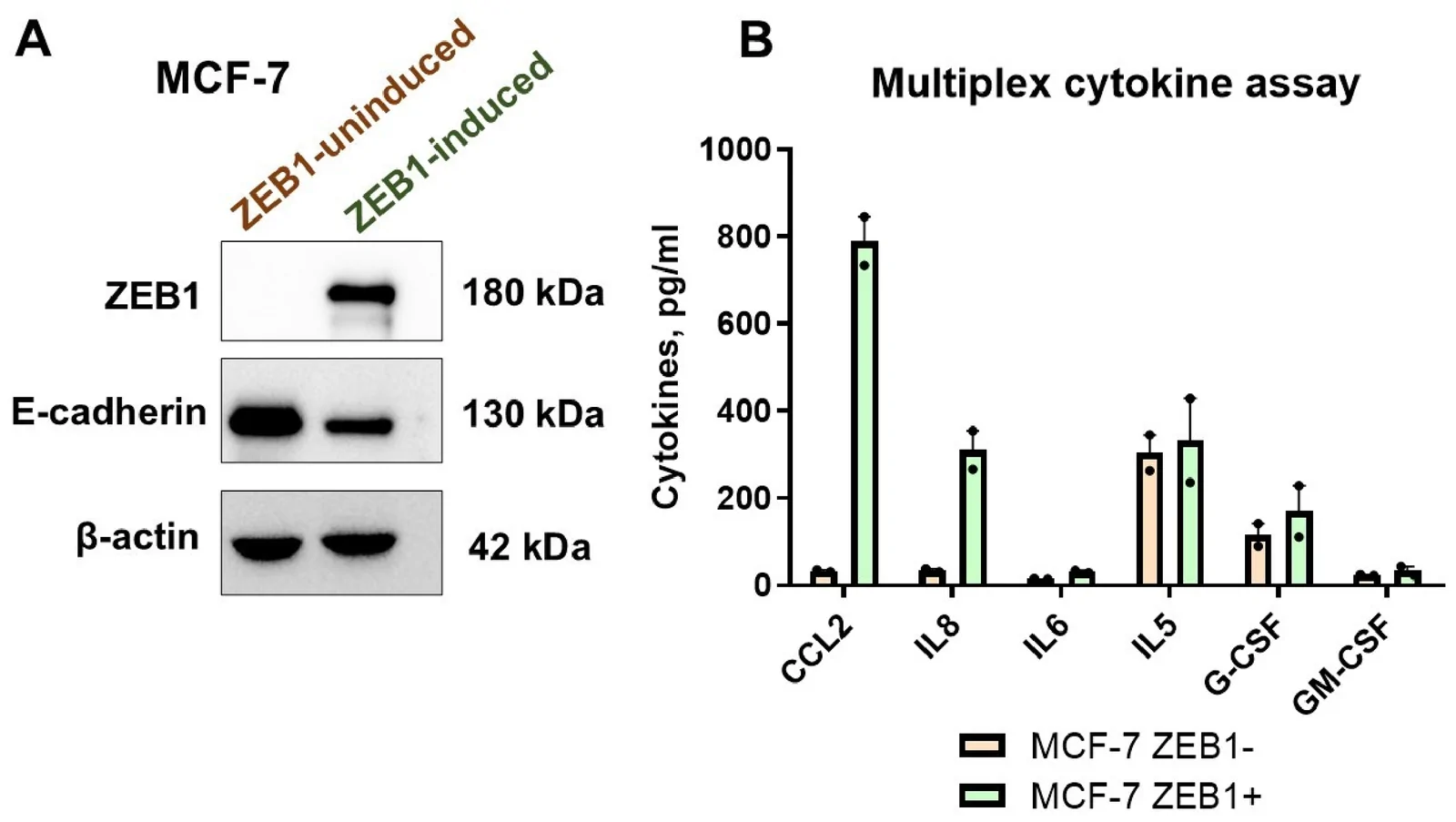
Effects of ZEB1 status on the expression of cytokines in MCF-7 cells. (**A**) Western blot of MCF-7 cells before and after ZEB1 induction. ZEB1-positive status correlates with a decreased level of the epithelial marker E-cadherin. (**B**) Cytokine profiling using the Bio-Plex Pro Assays kit (Bio-Rad, USA) revealed low levels of multiple cytokines in the conditioned medium of MCF-7 cells. Yet, the CCL2 chemokine level increased dramatically upon induction of ZEB1 expression. Data are presented as mean ± SD (*n* = 2).

To search for cytokines whose expression strictly depends on ZEB1, we used an already established MCF-7 cell system with Tet-inducible expression of ZEB1 [38]. Being epithelial, MCF-7 cells express E-cadherin but lack the expression of endogenous EMT factors, including ZEB1, providing a clean background. Doxycycline treatment of MCF-7-Tet-on cells caused a detectable induction of ZEB1 expression (Figure 1A). Western blot analysis demonstrated that doxycycline-induced activation of ZEB1 expression led to E-cadherin downregulation (Figure 1A). The conditioned media from MCF-7 cells with different statuses of ZEB1 expression were analyzed using a multiplex enzyme immunoassay. The profiling analysis revealed a low expression level of cytokines involved in the regulation of the tumor immune microenvironment in MCF-7 cells (Figure 1B). The concentrations of many cytokines including IL1-β, IL2, IL4, IL7, IL10, IL12, IL13, IL17, CCL4, IFN-γ, and TNF-α were below the level of detection (Supplementary Table S7). Nevertheless, the positive ZEB1 status correlated with the dramatic increase in the IL8 and primarily CCL2 protein concentrations in the conditioned medium (Figure 1B). The data published earlier confirms our results on ZEB1-dependent upregulation of IL8 in breast cancer cells. Thus, we decided to focus on studying the influence of ZEB1 on CCL2 expression.

### 2.2. ZEB1 Significantly Affects the CCL2 Expression in Response to TNF-α Treatment in MCF-7 and MDA-MB-231 Cell Lines

**Figure 2:**
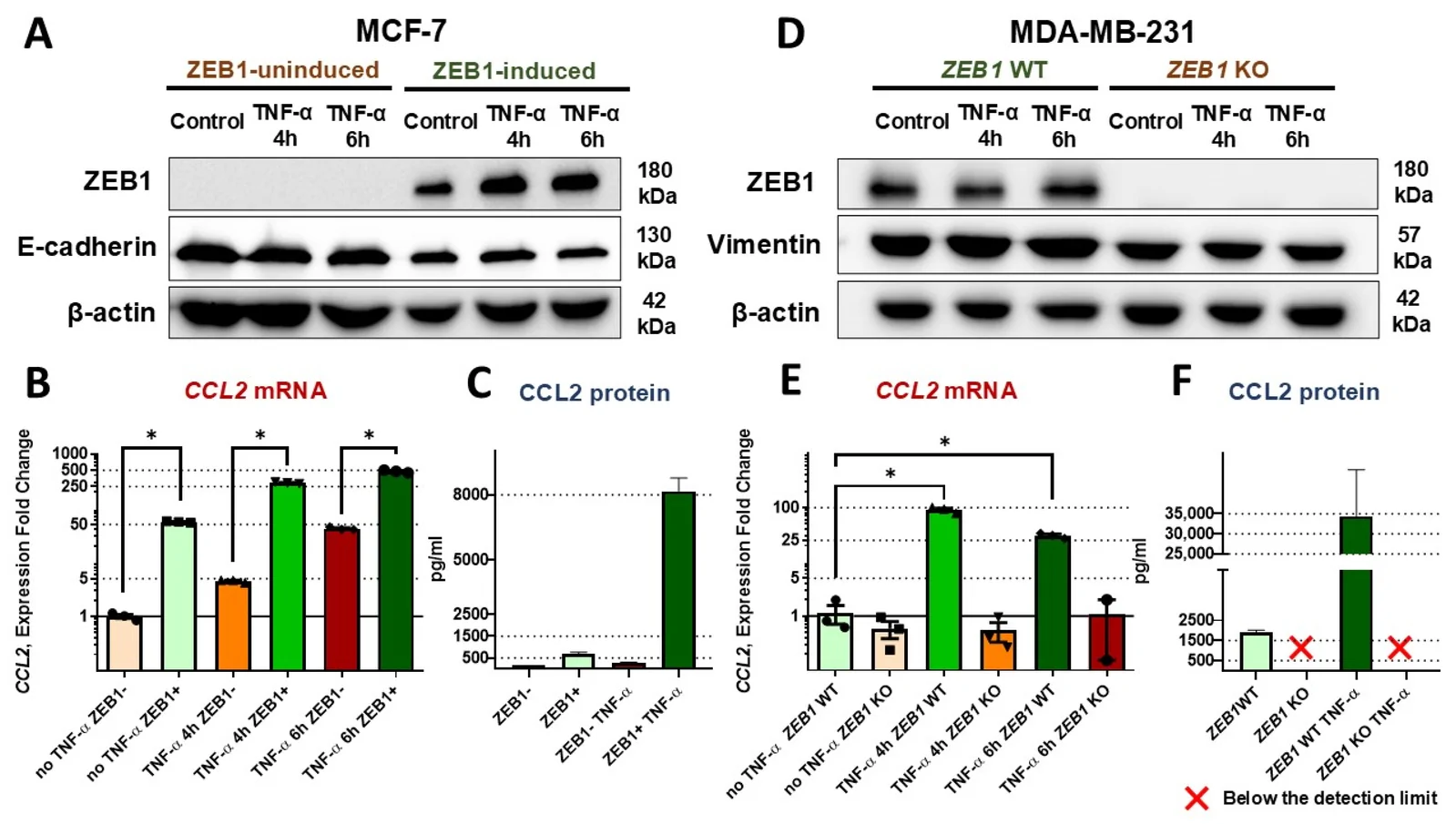
Synergistic effect of TNF-α treatment and ZEB1 on CCL2 expression in MCF-7 and MDA-MB-231 cells. (**A**,**D**) Western blots of ZEB1 and its targets, E-cadherin in MCF-7 Tet-on-ZEB1+/− (**panel A**) and Vimentin in MDA-MB-231 ZEB1+/− cells (**panel D**), after incubation with TNF-α (50 ng/mL). (**B**) RT-PCR of *CCL2* mRNA levels in MCF-7-ZEB1+/− cells after TNF-α treatment (*n* = 3). (**C**) CCL2 protein levels in MCF-7 Tet-on-ZEB1+/− cells incubated with TNF-α were determined by ELISA assay (*n* = 2). MDA-MB-231 cells with different statuses of ZEB1 (*ZEB1* WT vs. *ZEB1* KO) exhibit different levels of CCL2 expression following TNF-α treatment at the mRNA (*n* = 3) (**E**) and protein (*n* = 2) (**F**) levels, respectively. Protein concentrations below the ELISA detection level are indicated. All data are presented as mean ± SD. Statistical significance for RT-PCR data was assessed using one-way ANOVA and Tukey adjustment for multiple comparisons. * *p* < 0.05.

Because we found a positive correlation between ZEB1 and CCL2, which is the pivotal chemokine for recruitment of monocytes and macrophages, we wanted to explore this regulatory axis further. It is well established that monocytes and macrophages are the primary source of Tumor Necrosis Factor-alpha (TNF-α) secretion [39]. Thus, we sought to assess the functional significance of elevated TNF-α for ZEB1-mediated expression of CCL2 in different breast cancer cell lines. To this end, we treated MCF-7 cells with Tet-on ZEB1 expression and triple-negative breast cancer MDA-MB-231 cells bearing *ZEB1* gene knock-out with TNF-α (50 ng/mL) for different time periods. The respective parental cell lines without and with ZEB1 expression (MCF-7-ZEB1-Tet-on without induction and MDA-MB-231 with endogenously expressed *ZEB1*) were used as controls (Figure 2A,D). In the case of MCF-7 cells, induction of ZEB1 expression increased *CCL2* mRNA expression 50-fold (Figure 2B). The exposure of these cells to TNF-α for 4 h led to a 5-fold increase in *CCL2* expression for both ZEB1-negative and -positive cells without altering the ratio in *CCL2* expression between them. After 6 h of TNF-α treatment, the difference in the *CCL2* mRNA levels increased to 100-fold between cells with different ZEB1 statuses (Figure 2B). The primer sequences are provided in Table 1 (Materials and Methods, Section 4.4). The results of ELISA (Figure 2C) confirmed the RT-PCR data and demonstrated a significant increase in the CCL2 protein levels in conditioned medium with TNF-α-treated ZEB1-positive MCF-7 cells in comparison to ZEB1-negative MCF-7 cells. The more pronounced effect of the ZEB1 status on CCL2 was observed in MDA-MB-231 cells. These cells bearing the knocked-out *ZEB1* gene (Figure 2D) completely lost the ability to express CCL2 in response to TNF-α treatment both at the mRNA (Figure 2E) and protein levels (Figure 2F). Therefore, the ZEB1 status was crucial for CCL2 chemokine production in luminal type A and triple-negative breast cancer cell lines. Additionally, we measured the mRNA expression level of *IL6* and *CXCL8* (IL8) genes in these cell lines under the conditions described above. The results (Supplementary Figure S1) also showed the reinforcing effect of ZEB1-positive status on TNF-α-mediated transcriptional activation of pro-inflammatory genes that is consistent with published data [37].

### 2.3. THP-1 Macrophages Enhance ZEB1-Mediated MCF-7 Migration Capacity in Co-Culturing Conditions

**Figure 3:**
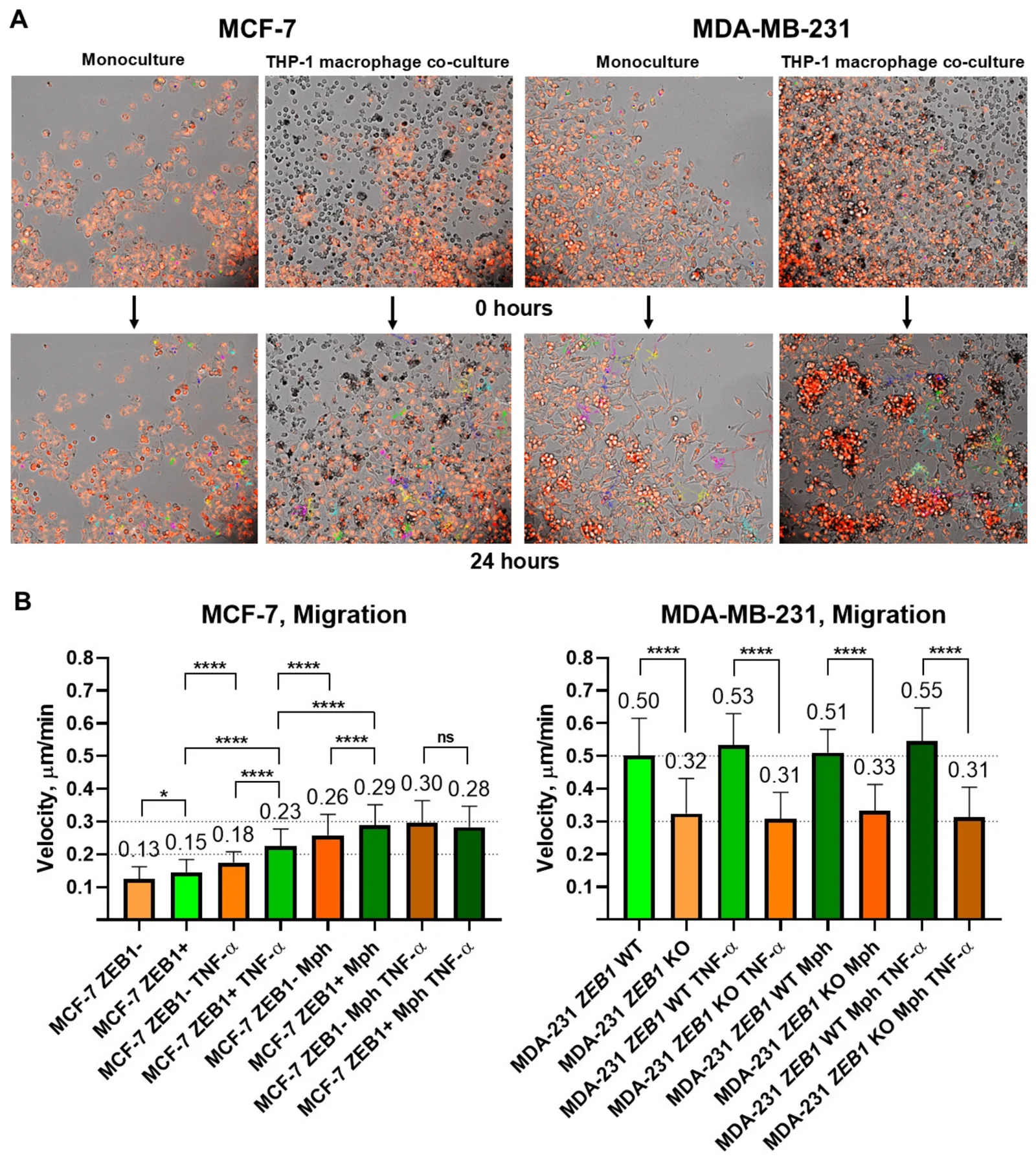
Cell tracking assay results on MCF-7 and MDA-MB-231 cells with different ZEB1 expression levels. (**A**) PKH26-labeled breast cancer cells were tracked over a 24 h period with a 15 min time-lapse interval. (**B**, **left panel**) Tet-on-induced ZEB1 expression in MCF-7 cells significantly enhances their motility. TNF-α also increases the average velocity of MCF-7-Tet-on-ZEB1 cells and further amplifies the upregulation of ZEB1-dependent migration. Co-culture with macrophages significantly enhances the motility of MCF-7 cells. (**B**, **right panel**) The migratory activity of MDA-MB-231 cells depends on ZEB1 status and is not significantly affected by TNF-α treatment or co-culture with macrophages. For each experimental group (defined by cell type, ZEB1 status, and TNF-α treatment or co-culture), three fields of view were analyzed, yielding 150 individual cell tracks per group. Data are presented as mean ± SD (*n* = 150 tracks per group). Statistical significance was assessed using one-way ANOVA with Tukey’s adjustment for multiple comparisons. * *p* < 0.05; **** *p* < 0.0001. Colors in the graphs are used for visual clarity only.

Based on our finding that CCL2 expression is regulated by both ZEB1 and TNF-α, we next investigated the reciprocal interactions between breast cancer cells with different ZEB1 statuses and macrophages under co-culture conditions. To this end, we performed live-cell imaging of MCF-7 and MDA-MB-231 cells with different ZEB1 statuses for 24 h using a 15 min time-lapse interval. Breast cancer cells were labeled with PKH26, whereas THP-1-derived macrophages were labeled with Hoechst dye. Using Fiji, we first assessed the motility of breast cancer cells in monoculture and co-culture by tracking individual cells and calculating their average velocity (Figure 3A).

As shown in Figure 3B and Supplementary Videos S1–S8, MCF-7 cells exhibited increased motility in the presence of ZEB1, consistent with the established role of ZEB1 as a driver of EMT and cell migration [40]. Notably, co-culture with macrophages markedly increased the velocity of both ZEB1-positive and ZEB1-negative MCF-7 cells. Consequently, following TNF-α treatment, the difference in motility between ZEB1-positive and ZEB1-negative MCF-7 cells was no longer apparent under co-culture conditions. In contrast, the velocity of MDA-MB-231 cells was unaffected by either TNF-α treatment or macrophage co-culture. Rather, their migratory capacity was strongly dependent on ZEB1 status, with ZEB1 knockout resulting in a pronounced reduction in cell velocity (Figure 3B; Supplementary Videos S9–S16).

Collectively, these findings suggest that ZEB1 may regulate cancer cell motility through both cell-intrinsic and microenvironment-dependent mechanisms. In MDA-MB-231 cells, ZEB1 directly contributes to the maintenance of high migratory activity, whereas in MCF-7 cells, ZEB1 may additionally participate in a positive feedback mechanism in which ZEB1-associated CCL2 production promotes macrophage recruitment, and macrophages, in turn, enhance the motility of neighboring ZEB1-negative cancer cells.

### 2.4. THP-1 Macrophages Form Aggregates with Breast Cancer Cells

**Figure 4:**
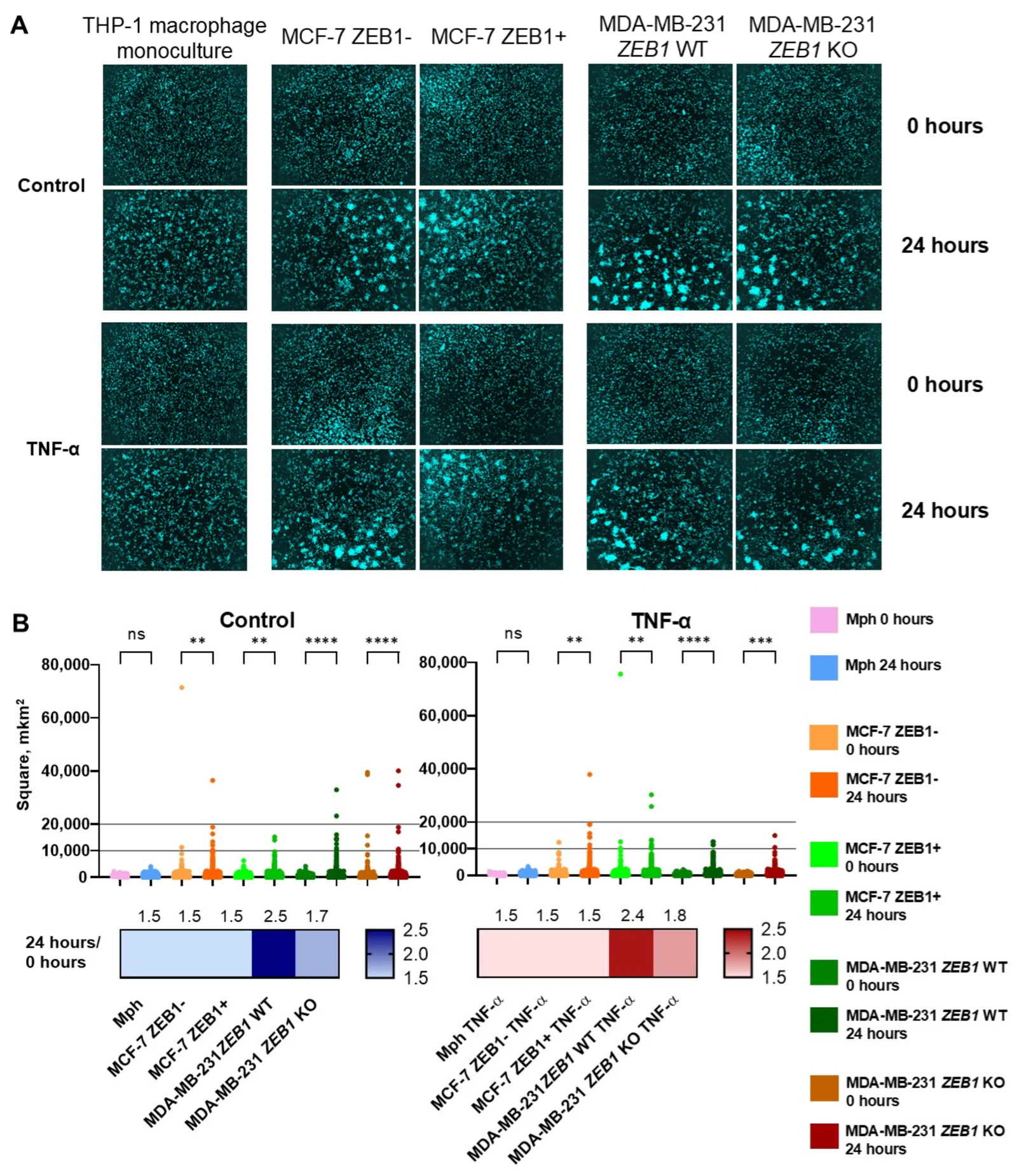
The macrophage aggregation process in the presence of breast cancer cells with different ZEB1 statuses. (**A**) Images of Hoechst-dye-labeled THP-1 macrophages at 0 h and 24 h of co-culture. (**B**) Distribution of macrophage aggregates by size before and after co-culture with MCF-7 (**left panel**) and MDA-MB-231 (**right panel**) cells. THP-1 macrophages in monoculture do not develop any detectable structures after 24 h. In contrast, co-culture conditions led to intensive macrophage aggregation. Nonetheless, no statistically significant differences were revealed for the size of aggregates at the 24 h time point with respect to the exact type of co-cultured breast cancer cells. However, the size difference between average macrophage aggregates at 0 h and 24 h was the highest under co-culture with MDA-MB-231 *ZEB1* WT cells. Three fields of view were analyzed per experimental group, and the total number of individual aggregates analyzed across these three fields ranged from 1500 to 3000 per group. Statistical significance was assessed using one-way ANOVA with Tukey’s adjustment for multiple comparisons. ** *p* < 0.01; *** *p* < 0.001; **** *p* < 0.0001; ns (not significant).

We next assessed the ability of THP-1-derived macrophages to aggregate during 24 h of co-culture. The number of objects detected in the blue (405 nm) channel was quantified at the beginning and end of the incubation period for THP-1 macrophages maintained in monoculture or co-culture with breast cancer cells (Figure 4A; Supplementary Figures S2 and S3). Macrophages cultured alone did not form large aggregates, irrespective of TNF-α treatment (Figure 4B). In contrast, extensive macrophage aggregation was observed in all co-culture conditions (Figure 4B). Notably, the ZEB1 status of the breast cancer cells did not significantly affect the area of macrophage aggregates at 24 h. Consistent with this observation, in co-cultures with MCF-7 cells, the ratio of aggregate area at 24 h to that at 0 h was comparable between ZEB1-negative and ZEB1-positive cells.

In contrast, macrophages co-cultured with MDA-MB-231 *ZEB1 WT* cells exhibited a markedly greater capacity to rearrange and form larger structures over the course of the experiment than macrophages co-cultured with MDA-MB-231 *ZEB1 KO* cells, as reflected by the significantly higher ratio of average object area at 24 h to that at 0 h (Figure 4B, lower graph). These findings suggest that the ZEB1 status of tumor cells can influence macrophage organization and interactions within the tumor cell–macrophage microenvironment. In particular, tumor cells with active ZEB1 signaling may promote more dynamic macrophage rearrangement and aggregation, potentially facilitating reciprocal interactions that contribute to tumor aggressiveness. Conversely, macrophage organization appears to be more strongly dependent on ZEB1 status in highly mesenchymal/metastatic MDA-MB-231 cells. This may suggest that ZEB1 remains an important determinant of tumor–immune cell interactions even after extensive loss of epithelial characteristics.

### 2.5. Conditioned Medium of TNF-α-Treated ZEB1-Positive Breast Cancer Cells Attracts THP-1 Macrophages

**Figure 5:**
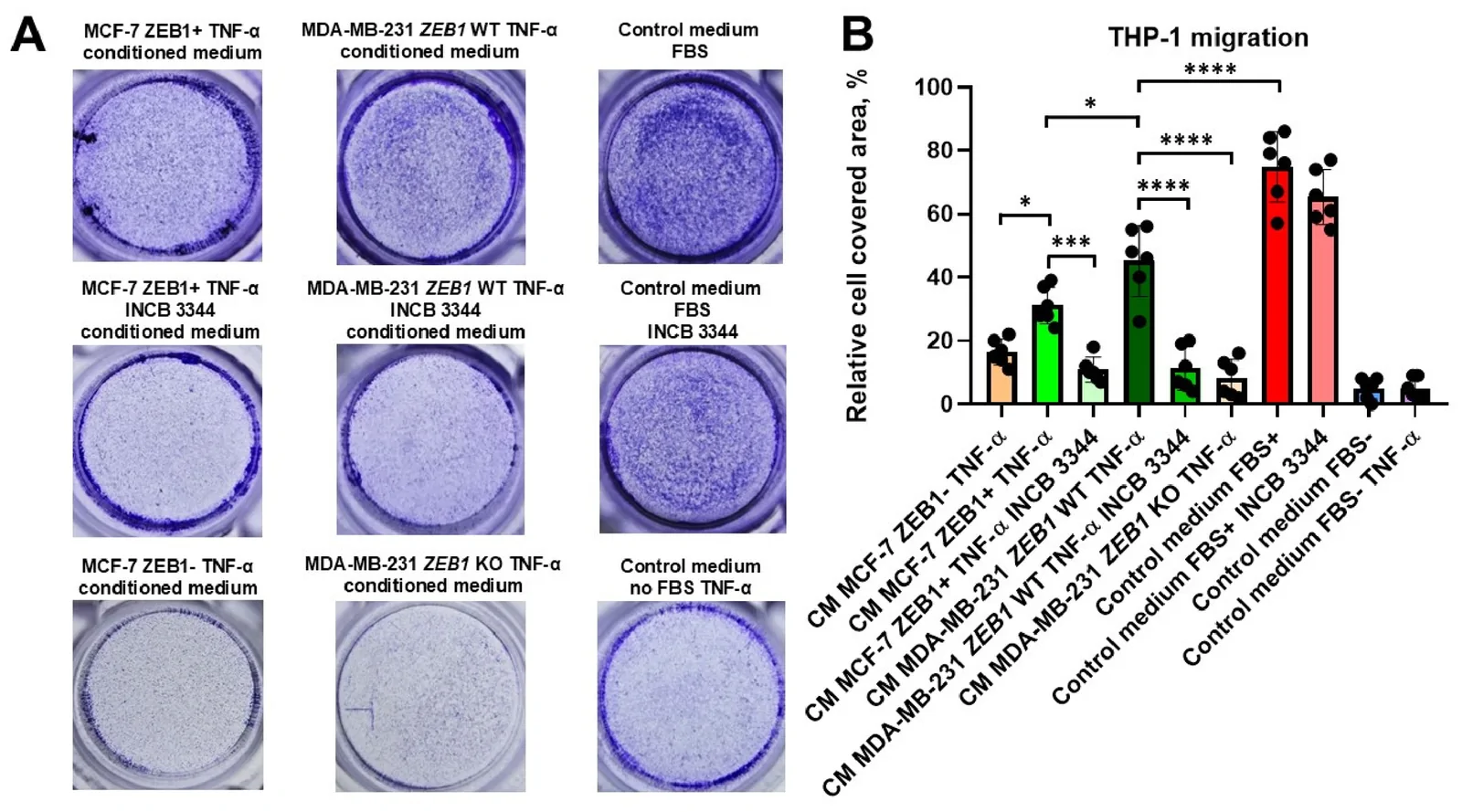
The Transwell migration assay of THP-1 macrophages. (**A**) Images of the bottom parts of inserts after 72 h of migration. (**B**) The graph shows the percentage of the area covered by stained macrophages on a lower surface of the inserts. Conditioned medium from TNF-α-treated ZEB1-positive MCF-7 and MDA-MB-231 cells enhanced macrophage migration, whereas the addition of INCB 3344 (CCR2 inhibitor) completely abolished this effect. Data presented as mean ± SD (*n* = 6). Statistical significance was determined by one-way ANOVA and Tukey adjustment for multiple comparisons, * *p* < 0.05; *** *p* < 0.001; **** *p* < 0.0001. Colors in the graph are used for visual clarity only.

To demonstrate the functional significance of ZEB1-dependent production and secretion of CCL2 by breast cancer cells in the chemoattraction of THP-1 macrophages, we performed a Transwell migration assay. Conditioned medium from TNF-α-treated MCF-7 and MDA-MB-231 cells was added to the lower chamber below the insert, while a suspension of macrophages was added to the upper part of the Transwell. After 72 h, the inserts were stained with crystal violet, and the cells remaining on the upper surface were removed by gentle wiping. Figure 5A shows images of the bottom side of the inserts. The medium from ZEB1-positive MDA-MB-231 and MCF-7 cells attracted significantly more macrophages than that from their ZEB1-negative counterparts (Figure 5A,B). Furthermore, treatment with the CCR2 receptor inhibitor INCB 3344 (100 nM) markedly attenuated macrophage migration toward conditioned medium from ZEB1-positive cells. The level of migration decreased to values comparable to those of control wells containing serum-free medium (Figure 5A,B). Notably, the strongest chemoattractive effect was exerted by complete medium containing FBS, and this effect was not significantly reduced by the addition of INCB 3344. These results confirm the crucial role of the CCR2–CCL2 axis in the recruitment of macrophages to ZEB1-positive breast cancer cells.

### 2.6. ZEB1 Potentially Interacts with CCL2 Gene Regulatory Elements

**Figure 6:**
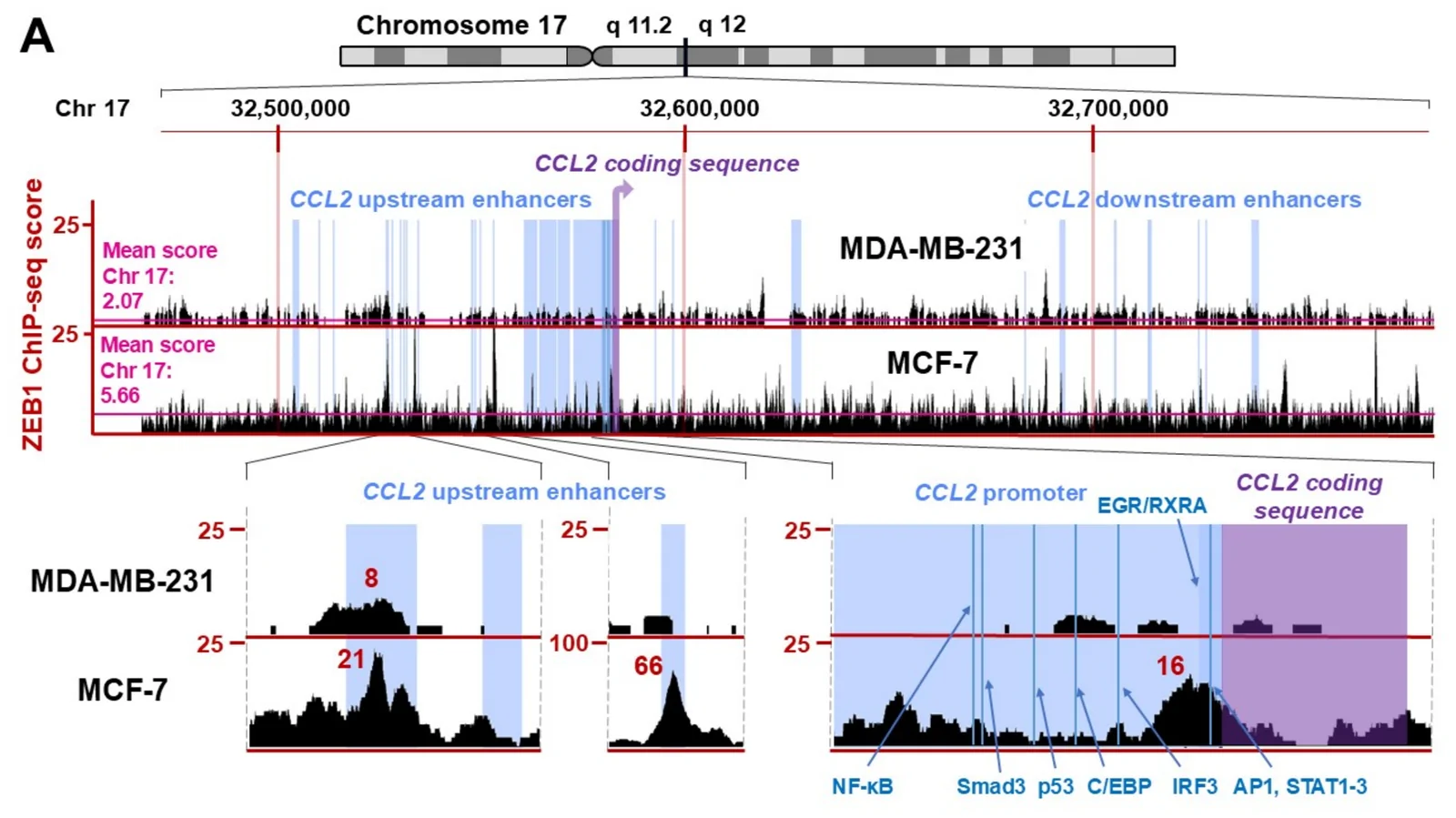

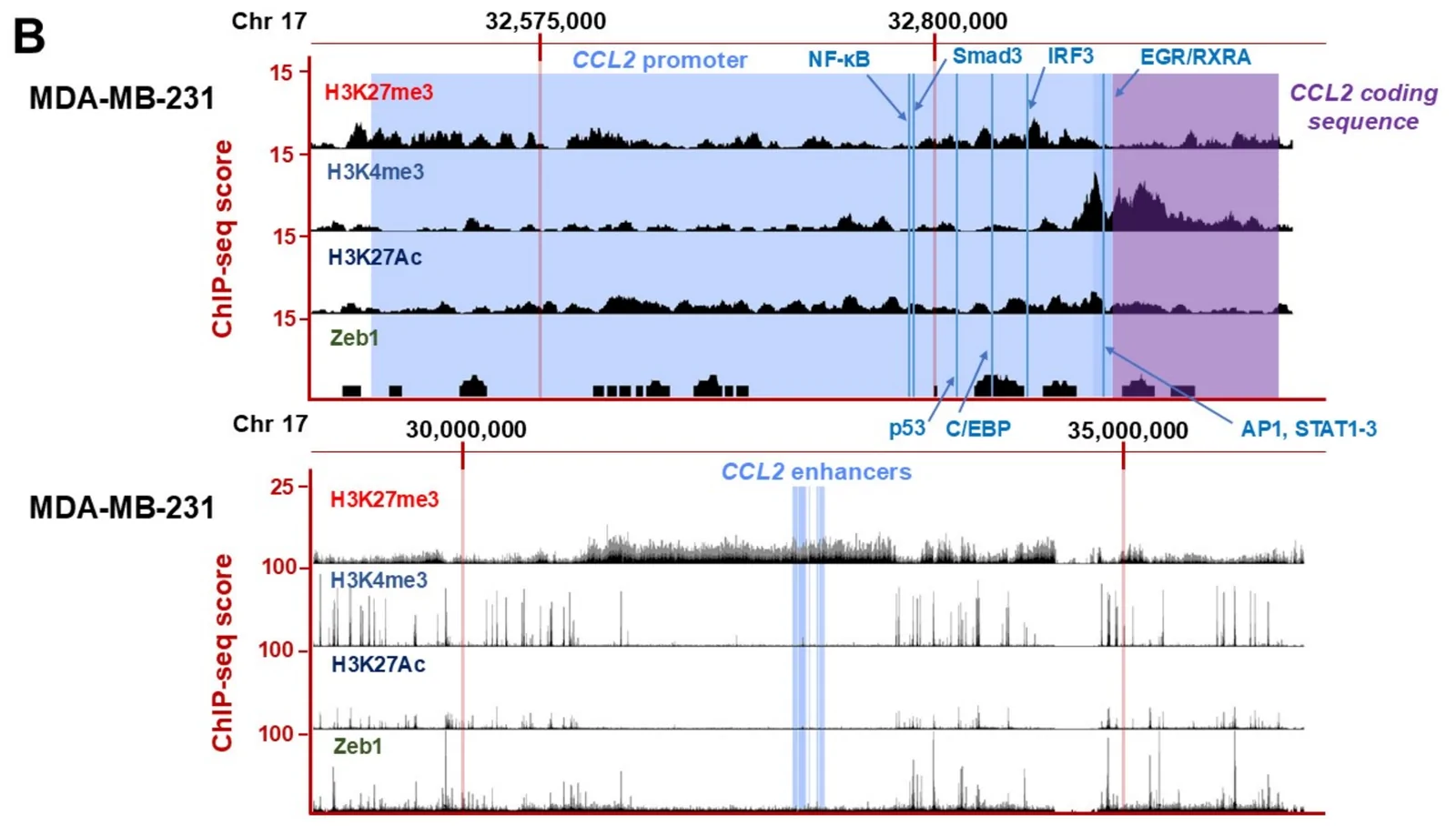
Visualization of ChIP-seq data for ZEB1 and chromatin regulating histone modifications in breast cancer cells using the UCSC Genome Browser (in GRCh37/hg19). (**A**) The ZEB1-ChIP-seq tracks in MCF-7 (with inducible ZEB1 expression) and MDA-MB-231 cells. The *CCL2* coding sequence is depicted in purple, whereas the enhancers and promoter are marked in blue. The lower panel represents the *CCL2* gene regulatory elements. ZEB1-enriched peaks that significantly exceed the mean ChIP-score for chromosome 17 are indicated. The bottom right image represents the *CCL2* gene promoter including the sites recognized by several transcription factors mentioned in articles. (**B**) The structure of the *CCL2* gene and its regulatory elements based on the UCSC Genome Browser visualization in MDA-MB-231 cells. The ChIP-seq tracks for ZEB1, H3K4me3, H3K27Ac and H3K27me3 are shown. The upper and lower panels show the same chromosome 17 region at different magnification levels.

To further evaluate the potential role of ZEB1 in regulating CCL2 expression, we analyzed publicly available ChIP-seq datasets from breast cancer cell lines. The *CCL2* promoter and putative enhancer regions were defined based on GeneCards annotations in the UCSC Genome Browser (GRCh37/hg19) (Figure 6A,B). We then examined ZEB1 ChIP-seq profiles from MDA-MB-231 cells (GSM2360622) and MCF-7 cells with inducible ZEB1 expression (GSE190095). Several upstream regulatory elements within the *CCL2* locus displayed ZEB1 ChIP-seq signals above the mean signal level across chromosome 17, suggesting potential involvement in ZEB1-dependent regulation of *CCL2* expression. The first element, GeneHancer ID GH17J034199, is shown at higher magnification in the bottom-left panel of Figure 6A. This element exhibited the highest ZEB1-binding signal among the analyzed *CCL2* enhancers in MDA-MB-231 cells and also showed a distinct ZEB1 signal above the mean level in MCF-7 cells, suggesting that it may contribute to *CCL2* regulation in both cell lines. A second upstream enhancer, GH17J034226, contained a prominent local maximum of the ZEB1 ChIP-seq signal in MCF-7 cells, substantially exceeding the mean signal level (Figure 6A, bottom-center panel). In contrast, no comparable ZEB1 enrichment was observed at this site in MDA-MB-231 cells.

The *CCL2* promoter is associated with the GeneHancer element GH17J034245. Its promoter-proximal region contains binding sites for several reported *CCL2* transcriptional regulators (dark-blue lines) [41] (Figure 6A, bottom-right panel). Notably, the two cell lines exhibited distinct ZEB1-binding patterns. In MDA-MB-231 cells, ZEB1 enrichment was almost absent at the site closest to the transcription start site, whereas MCF-7 cells showed a clear increase in ZEB1 ChIP-seq signal in this region. The adjacent upstream sequence contains predicted recognition motifs for STAT1 and STAT3 [42], EGR/RXCR [43], and the AP-1 complex [44]. Thus, the relatively strong ZEB1 enrichment at several enhancer elements and near the *CCL2* promoter in MCF-7 cells is consistent with a potential role for ZEB1 in facilitating *CCL2* transcription, particularly under inflammatory conditions.

This interpretation is further supported by previous evidence that ZEB1 interacts with AP-2 in MCF-7 cells and contributes to the maintenance of an active chromatin state at target gene loci [45]. However, our experimental data argue against a major contribution of AP-1 signaling to CCL2 induction in the present model because Western blot analysis failed to detect c-Jun or phosphorylated c-Jun in MCF-7 cells. This result suggests that AP-1 activation is unlikely to account for the observed increase in CCL2 expression. In contrast, phosphorylated STAT1 levels were elevated in response to TNF-α treatment in both ZEB1-positive and ZEB1-negative MCF-7 cells (Supplementary Figure S4). These findings raise the possibility that ZEB1 contributes to maintaining a permissive chromatin environment at the *CCL2* locus, thereby facilitating access to various inflammatory transcription factors such as STAT1 in response to environmental cues, e.g., TNF-α, to promote *CCL2* transcription.

The analysis of MDA-MB-231 cells provides a different picture, suggesting the possibility of a ZEB1-indirect effect on *CCL2* transcription activation. Importantly, MDA-MB-231 cells express relatively high levels of CCL2, which was completely lost following *ZEB1* knockout, suggesting that ZEB1 may be required to maintain the chromatin state necessary for *CCL2* transcription. To investigate this possibility, we analyzed publicly available ChIP-seq datasets for H3K4me3 (GSM2862182) and H3K27ac (GSM2862183), established markers of active chromatin, together with H3K27me3 (GSM2862180), a marker of transcriptionally repressive chromatin (Figure 6B).

The *CCL2* regulatory region was located within an area of relatively high H3K27me3 enrichment, although a modest local maximum of H3K4me3 was evident around the promoter. Western blot analysis revealed no substantial activation of the examined inflammatory signaling pathways in ZEB1-positive MDA-MB-231 cells following TNF-α treatment. Phosphorylated STAT1, c-Jun, and phosphorylated c-Jun were undetectable, whereas phosphorylated STAT3 was reduced following TNF-α treatment (Supplementary Figure S4). NF-κB, another major pro-inflammatory transcriptional regulator, exhibited ZEB1-dependent protein levels under basal conditions; however, this difference was no longer apparent following TNF-α treatment. Thus, although NF-κB activity may contribute to the ZEB1-dependent regulation of CCL2 under basal conditions, its activation by TNF-α appears to be largely independent of ZEB1.

Taken together, these observations suggest that, in MDA-MB-231 cells, ZEB1 may be required to maintain a permissive chromatin configuration at the *CCL2* locus, thereby allowing basal and/or stimulus-dependent transcription. Loss of ZEB1 may consequently promote a shift toward a more repressive chromatin state that limits the ability of pro-inflammatory signaling pathways to induce CCL2 expression.

Collectively, these findings support a model in which ZEB1 contributes to CCL2 regulation through mechanisms that are at least partly cell-type-dependent. In MCF-7 cells, ZEB1 enrichment at the *CCL2* promoter and upstream enhancers is consistent with a role in maintaining a transcriptionally permissive chromatin environment that facilitates the activity of inflammatory transcription factors such as STAT1. In MDA-MB-231 cells, by contrast, the absence of strong ZEB1 enrichment at the promoter argues against direct transcriptional activation by promoter-bound ZEB1. Instead, we hypothesize that ZEB1 may contribute to the maintenance of an active or permissive chromatin state at the *CCL2* locus. Accordingly, loss of ZEB1 is associated with acquisition of a more repressive chromatin configuration and complete suppression of CCL2 expression, consistent with previous observations [46].

### 2.7. The Patient Datasets Demonstrate Correlation Between CCL2 and ZEB1 Expression for Some Breast Cancer Subtypes

**Figure 7:**
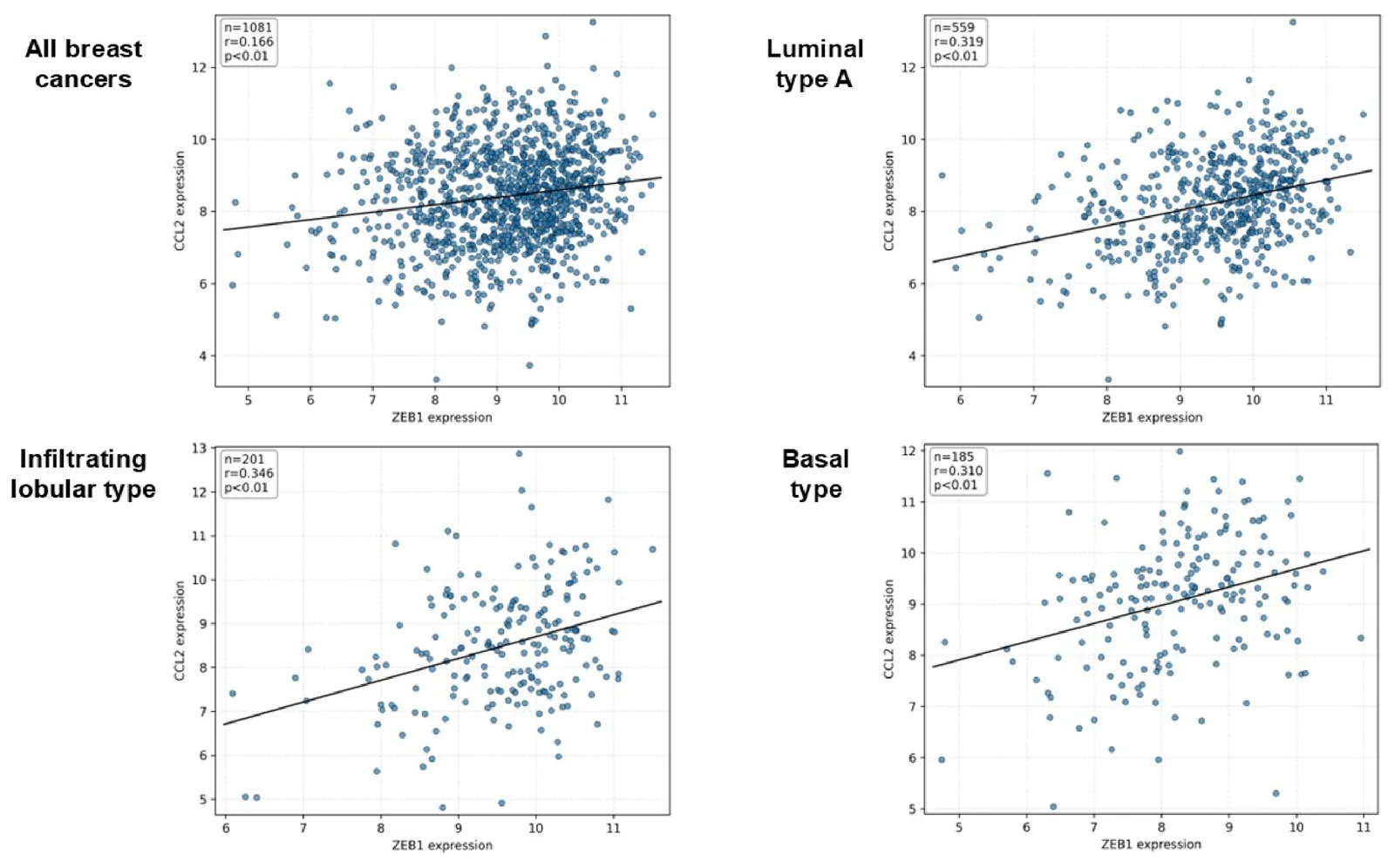
Analysis of Pearson’s correlation between the expression of ZEB1 and CCL2 genes in breast cancer datasets. Aggregated data encompassing all breast cancer variants indicates no correlation between ZEB1 and CCL2 expression levels. Datasets for luminal type A, basal type and infiltrating lobular type exhibit a moderate connection for the specified genes. The format employed is log2(RSEM + 1) axes.

To determine whether the link between ZEB1 and CCL2 expression observed in vitro can be recapitulated in the clinical setting, we studied the breast cancer patient data from the TCGA Pan-Cancer Atlas (PanCanAtlas) project (the detailed data analysis protocol is described in the Section 4). Pearson’s correlation analysis revealed no significant correlation between ZEB1 and CCL2 expression levels when all types of breast cancer were used for analysis (Figure 7). However, when we analyzed this correlation using specific subtypes, e.g., luminal type A, infiltrating lobular type, or basal type groups, a medium-strength correlation was noted in all three sub-groups (Figure 7). Furthermore, a comparable correlation (*r* = 0.336, *n* = 151) was revealed in the group of patients with lethal outcome (see Supplementary Figure S5). Other groups of patients demonstrated no correlation between ZEB1 and CCL2 or had a small sample size (see Supplementary Figure S5). The concurrent upregulation of these genes in luminal type A and infiltrating lobular type groups is consistent with the results obtained using MCF-7-ZEB1-Tet-on cells. Collectively, these bioinformatic findings support the relevance of the ZEB1–CCL2 association in specific breast cancer contexts.

## 3. Discussion

ZEB1 drives molecular programs that reshape the cancer cell phenotype and promote the dissemination of malignant clones to distant sites. It further modulates the crosstalk between cancer cells and the immune microenvironment. The interaction between ZEB1-positive cancer cells and tumor-associated macrophages (TAMs) represents one of the key mechanisms influencing cancer spread and disease progression. The pivotal role of ZEB1 in mediating interactions between cancer cells and TAMs has previously been demonstrated in melanoma [47], lung [48], and prostate cancers [49]. In the present study, we used mammary carcinoma cell lines and THP-1-derived macrophages as in vitro models to investigate the effect of ZEB1 on the ability of breast cancer cells to recruit and interact with tumor-associated macrophages (TAMs), whose contribution to breast cancer progression is well established [50,51]. Although THP-1-derived macrophages represent a simplified model compared with primary monocyte-derived macrophages, this cell line is widely used to study monocyte/macrophage chemotaxis and migratory behavior, making it a practical model for studying ZEB1-dependent interactions with TAMs [52,53].

To identify cell-context-independent mechanisms governed by ZEB1 in TAM recruitment, we sought to define the ZEB1-dependent cytokine profile in the breast epithelial cell line MCF-7, which was engineered to inducibly express ZEB1 upon addition of doxycycline to the culture medium [16] (Figure 1). Among several cytokines that showed robust dependence on ZEB1 were CCL2 and IL-8 (Figure 1B). Our finding that ZEB1 enhances IL-8 expression is consistent with previously published data by Katsura et al. [37], who demonstrated upregulation of IL-6 and IL-8 in several triple-negative breast cancer (TNBC) cell lines. Surprisingly, those authors did not identify CCL2 as a ZEB1 target in TNBC cells, which may reflect the fact that we employed epithelial MCF-7 cells lacking endogenous ZEB1 expression, where the cytokine spectrum may differ from that of cells that have undergone EMT. These observations prompted us to examine the relationship between ZEB1 and CCL2 in mesenchymal cells with endogenous ZEB1 expression. MDA-MB-231 cells lacking ZEB1 (*ZEB1* KO) exhibited a full loss of ability to synthesize CCL2 chemokine (Figure 2F). To enhance potential effects of ZEB1 on CCL2 expression, we treated the cells with the pro-inflammatory cytokine TNF-α, which is secreted by macrophages, as several studies have demonstrated its stimulatory effect on ZEB1-dependent EMT [54,55,56,57,58]. TNF-α treatment amplified dramatically the level of CCL2 protein expression in the parental MDA-MB-231 cell line, although it failed to affect the CCL2 production in ZEB1-negative cells. Importantly, our results strongly suggest that TNF-α-mediated upregulation of CCL2 is not linked to increased ZEB1 expression, as ZEB1 levels remained unchanged after TNF-α exposure (Figure 2D). Given the pleiotropic effects of TNF-α [59], we hypothesize that this response may involve induction of additional transcription factors, such as Sp1 and NF-κB, which are known regulators of the *CCL2* gene [60,61]. As expected, the results of our Transwell migration assay confirmed that ZEB1-mediated CCL2 secretion facilitated THP-1 macrophage recruitment, supporting our hypothesis that the ZEB1–CCL2 axis is instrumental in recruiting TAMs to cancer cells at the invasive tumor front (Figure 5). It is also well established that TAMs markedly enhance the migratory and invasive capacity of cancer cells [62,63]. Consistent with this notion, co-culture of THP-1-derived macrophages with MCF-7 cells increased the migratory activity of the latter by at least threefold (Figure 3). In contrast, we did not observe a similar effect in mesenchymal MDA-MB-231 cells (Figure 3B, right panel). The only pronounced effect on MDA-MB-231 cell motility was determined by ZEB1 status. It may be speculated that macrophage-secreted cytokines, including IL-6, IL-8, and TGF-β, promote the onset of EMT in epithelial cells, thereby increasing their migratory and invasive potential [64]. Expression of ZEB1 alone may not be sufficient to fully reverse the epithelial phenotype of MCF-7 cells to a mesenchymal state, as forced ZEB1 expression only minimally affected cell motility. By contrast, mesenchymal MDA-MB-231 cells, which had already acquired the capacity for individual migration, were largely unresponsive to co-culture with macrophages in this regard. However, our results suggest that these cells still require ZEB1 for migration (Figure 3). Future studies should clarify the underlying mechanisms.

It should also be noted that ZEB1 can suppress expression of the pro-epithelial microRNA miR-34, which is highly expressed in p53-positive MCF-7 cells [65]. Substantial evidence in the literature indicates that miR-34 attenuates cancer cell motility [66].

The reciprocal effects of tumor cells on THP-1-derived macrophages were also examined (Figure 4). In vivo, macrophages can form granuloma-like structures around foreign cells to limit their dissemination throughout the body [67]. Furthermore, close spatial interactions between ZEB1^+^/CD47^+^/PD-L1^+^ cancer cells and M2 macrophages have been observed in lung cancer [68]. Macrophages have also been shown to coat cancer cells and protect them from immune attack [69]. In addition, Cortes et al. [70] demonstrated that TAM depended on ZEB1 expression for their tumor-promoting functions and the role of macrophage-produced CCL2 has been demonstrated for the malignant transformation of breast epithelial cells [71]. In vitro, we observed an increased tendency of macrophages to aggregate when incubated in the presence of tumor cells. Interestingly, macrophages tended to form larger aggregates in the presence of tumor cells lacking ZEB1, and this effect was observed with both MCF-7 and MDA-MB-231 cells (Figure 5). The addition of TNF-α to the medium reduced this tendency despite the increased concentration of CCL2 in the medium.

Our ChIP-seq analysis further supports a potential regulatory relationship between ZEB1 and CCL2. The identification of ZEB1 occupancy at regulatory regions within the *CCL2* locus suggests that CCL2 may represent a direct transcriptional target of ZEB1 in MCF-7 cells (Figure 6), consistent with the observed increase in CCL2 expression following ZEB1 induction. By contrast, in MDA-MB-231 cells, the complete loss of CCL2 expression following *ZEB1* knockout suggests that ZEB1 may regulate CCL2 indirectly by contributing to the maintenance of a permissive epigenetic state at the *CCL2* locus.

In this respect, it should be noted that in addition to ZEB1, other transcriptional factors also regulate the expression of CCL2, (e.g., Sp1 and others) [72]. These transcription factors are also responsive to TNF-α treatment and may substitute ZEB1 for activation of CCL2.

What is the functional significance of ZEB1-enhanced recruitment of TAMs to breast cancer cells? First, ZEB1 upregulation under conditions of metabolic reprogramming may influence tumor-infiltrating macrophages [73]. For example, hypoxia has been shown to promote CD163^+^ macrophage recruitment via the ZEB1–CCL8 axis in a cervical cancer model [35]. Another study demonstrated that ZEB1-mediated glycolysis leads to lactate accumulation and subsequent M2 macrophage polarization, thereby promoting breast cancer progression [74]. In addition, CD47 has been identified as a direct transcriptional target of ZEB1 [75].

Conversely, cytokines produced by immune cells, including macrophages, can promote ZEB1-driven EMT. For example, IL1-β has been shown to activate ZEB1 in colon cancer cells [76]. However, another study demonstrated that IL1-β-mediated ZEB1 upregulation attenuates the reverse mesenchymal-to-epithelial transition and prevents secondary tumor formation [77]. In the case of TNF-α, multiple studies have demonstrated its role in regulating ZEB1-dependent EMT across different cancer types [55,56,57,58].

Overall, the prognostic significance of macrophage infiltration has been demonstrated across multiple cancer types [78,79,80,81], and the role of TAMs has been extensively reviewed [82,83,84,85]. Notably, there is currently no direct experimental evidence showing that ZEB1 upregulation in breast cancer cells promotes CCL2 expression and macrophage recruitment. Although the role of CCL2 in ZEB1-mediated therapy resistance has been reported in lung cancer [86], and ZEB1 has been shown to be important for macrophage function [87], a direct mechanistic link remains elusive. However, ZEB1-dependent CCL2 expression and subsequent macrophage infiltration have been demonstrated in trophoblast models [88]. Therefore, we undertook a bioinformatics approach to define the prognostic significance of ZEB1 and CCL2 co-expression with respect to the survival of breast cancer patients (Figure 7). Importantly, the most statistically significant correlation between ZEB1 and CCL2 was detected in basal-like cells, which suggests that at this advanced stage of cancer, a lot of cells already express ZEB1 and have undergone EMT [89,90].

Collectively, our data therefore complement existing studies by elucidating complex reciprocal interactions between malignant cells and macrophages. We demonstrate that ZEB1 plays a key role in regulating CCL2 expression in both luminal A and triple-negative breast cancer cells. The results obtained on MCF-7 ZEB1 Tet-on cells suggest a scenario in which specific malignant clones with an activated EMT program would alter the tumor microenvironment by recruiting monocyte-derived macrophages. Consequently, these macrophages may further affect neighboring epithelial-like cancer cells by promoting their migratory capacity, thus facilitating local invasion. As part of this feed-forward regulatory loop, elevated TNF-α levels within tumors may further amplify ZEB1-mediated effects. These findings are consistent with statistical analyses of breast cancer patient datasets, which reveal correlations between ZEB1 and CCL2 expression in specific patient subgroups.

Taken together, our findings identify the ZEB1–CCL2 axis as a potential molecular link between EMT and macrophage recruitment in breast cancer, with its contribution likely varying according to the cellular context and stage of tumor progression. Our findings also complement previous studies demonstrating the roles of other EMT transcription factors, such as Snail [34] and Twist1 [36] in regulating CCL2 expression.

## 4. Materials and Methods

### 4.1. Cell Lines

Breast cancer cell lines MCF-7 and MDA-MB-231 as well as acute monocytic leukemia cells, THP-1, were cultured at 37 °C in an atmosphere of 5% CO_2_. For breast cancer cells, the DMEM used contained 10% fetal bovine serum, L-glutamine (2 mM) and gentamicin (50 μg/mL). The THP-1 cells were cultured in RPMI medium also containing 10% fetal bovine serum, L-glutamine (2 mM), and gentamicin (50 μg/mL). The MCF-7 Tet-on^®^ line with inducible ZEB1 expression was generated by co-transfection of the rtTA) vector and the pBI vector (Clontech Laboratories, Inc., A Takara Bio Company, Mountain View, CA, USA) encoding ZEB1 with EGFP at the C-terminal fragment. Induction of ZEB1 expression was carried out by adding doxycycline (1 μg/mL) to the culture medium for 72 h.

To obtain MDA-MB-231 cells carrying a ZEB1 knock-out, Lipofectamine™ 2000 (Thermo Fisher Scientific, Waltham, MA, USA) transfection was performed using the CRISPR-Cas9 vector pRG2 carrying guide-RNA against ZEB1 (Addgene plasmid # 104174) [91].

### 4.2. TNF-α Treatment

The cells were treated with the human recombinant TNF-α (SCI-store, Moscow, Russia) for 4, 6 and 24 h (concentration, 50 ng/mL). Cells were washed with phosphate-buffered saline solution and the cultivation medium was replaced with serum-free RPMI before the cytokine addition.

### 4.3. Western Blot

Protein electrophoresis was performed using the Laemmli method. Samples containing cells were washed with phosphate-buffered saline to remove any residual media, after which cells were lysed in RIPA buffer (NaCl 150 mM; Nonidet P-40 1%; DOC 0.5%; SDS 0.1%; Tris (pH 7.4) 50 mM). The protein concentration was determined by the turbidimetric method using the Bradford reagent (Bio-Rad Laboratories, Inc., Hercules, CA, USA) by dissolving the samples 10 times with distilled water. Denaturation of proteins was carried out by heating the samples to 98 °C in Laemmli buffer (Tris–HCl (pH 6.8) 312.5 mM; SDS 10%; Glycerol 25%; Bromophenol blue 0.5%; 10% β-Mercaptoethanol) for 5 min. Sample separation was carried out in a polyacrylamide gel (acrylamide concentration, 10%) in Tris-glycine buffer (Bio-Rad, USA) containing 0.2% SDS at a voltage of 120 V. The proteins separated in the gel were transferred to a PVDF membrane (Merck Millipore (MilliporeSigma), Burlington, MA, USA) by the wet transfer method in Tris-glycine buffer with the addition of ethanol at a voltage of 100 V for 2 h. The membrane was incubated for 20 min in a 5% solution of dry milk in phosphate-buffered saline containing Tween-20, 1%. Incubation with primary antibodies was carried out overnight at 4 °C. To develop the signal, secondary goat anti-rabbit antibodies were used, conjugated with horseradish peroxidase and dissolved in phosphate-buffered saline with Tween-20, at a dilution of 1:10,000. The signal was recorded using the ChemiDoc gel documentation system (Bio-Rad, USA). We used primary antibodies for the following proteins: Actin (1:1000 dilution, β-Actin (13E5), Rabbit mAb #4970; Cell Signaling Technology, Inc., Danvers, MA, USA), ZEB1 (1:1000 dilution, ZEB1 (D80D3), Rabbit mAb #3396; Cell Signaling Technology, Inc., Danvers, MA, USA), E-cadherin (1:1000 dilution, E-Cadherin (24E10), Rabbit mAb #3195; Cell Signaling Technology, Inc., Danvers, MA, USAVimentin (1:1000 dilution, Mouse mAb sc-6260; Santa Cruz Biotechnology, Inc., Dallas, TX, USA), STAT1 (1:1000 dilution, STAT1 Rabbit mAb #9172; Cell Signaling Technology, Inc., Danvers, MA, USA), phospho-STAT1 (1:1000 dilution, Phospho-Stat1 (Tyr701) (58D6), Rabbit mAb #9167; Cell Signaling Technology, Inc., Danvers, MA, USA), c-Jun (1:1000 dilution, c-Jun Rabbit polyclonal Ab #9162; Cell Signaling Technology, Inc., Danvers, MA, USA), phospho-c-Jun (1:1000 dilution, Phospho-c-Jun (Ser63) II, Rabbit polyclonal Ab #9261; Cell Signaling Technology, Inc., Danvers, MA, USA), NF-κB p65 (1:1000 dilution, Rabbit polyclonal Ab, sc-372; Santa Cruz Biotechnology, Inc., Dallas, TX, USA), STAT3 (1:1000 dilution, Mouse mAb sc-293151; Santa Cruz Biotechnology, Inc., Dallas, TX, USA), and phospho-STAT3 (1:1000 dilution, Phospho-Stat3 (Tyr705) (D3A7), Rabbit mAb #9145; Cell Signaling Technology, Inc., Danvers, MA, USA).

### 4.4. RT-PCR

Total RNA was isolated using Extract-RNA reagent (Evrogen JSC, Moscow, Russia) according to the manufacturer’s protocol. cDNA synthesis was carried out using oligoDT primers (Evrogen JSC, Moscow, Russia) according to the manufacturer’s instructions. Real-time PCR was performed using a CFX96 Touch thermal cycler (Bio-Rad Laboratories, Inc., Hercules, CA, USA) using the qPCRmix-HS kit (Evrogen JSC, Moscow, Russia). To do this, 5x qPCRmix and forward and reverse primers of the gene of interest at a final concentration of 0.2 μM and 3 μL of cDNA were added to 20 μL of the reaction mixture. The amplification cycle began with 5 min at 95 °C, and then 40 cycles: 95 °C at 10 s, 63 °C at 15 s, and 72 °C at 15 s. Each experimental point was analyzed in triplicate. The expression level was calculated using the ΔΔCt method; the GAPDH gene was used as a reference control.

**Table 1:** Oligonucleotide sequences used in qPCR.

Gene	5′-3′ Primer Sequence
*GAPDH*	F GTCTCCTCTGACTTCAACAGCG R ACCACCCTGTTGCTGTAGCCAA
*CCL2*	F GCCTCCAGCATGAAAGTCTC R AGGTGACTGGGGCATTGAT
*IL6*	F GAACTCCTTCTCCACAAGCG R TCTGAAGAGGTGAGTGGCTGTC
*CXCL8* (IL8)	F GAGAGTGATTGAGAGTGGACCAC R CACAACCCTCTGCACCCAGTTT

### 4.5. Multiplex Cytokine Analysis

To select the conditioned medium for cytokine profile determination, the MCF-7 cells were planted in 6-well plates at a concentration of 100,000 cells/mL. Cells were cultured in RPMI medium not containing FBS. To sediment cellular debris, samples of the conditioned medium were centrifuged for 10 min at 2000× *g* and then stored at −80 °C. The analysis was performed using the Bio-Plex Pro Assays kit (Bio-Rad Laboratories, Inc., Hercules, CA, USA) in accordance with the manufacturer’s protocol on a MAGPIX System with an xPONENT 4.2 device (Merck & Co., Inc., Kenilworth, NJ, USA).

### 4.6. ELISA

To select the conditioned medium for ELISA, the breast cancer cells were planted at a concentration of 100,000 cells/mL. The content of the chemokine CCL2 in the conditioned medium of breast cancer cells was determined using a set of reagents for the enzyme-linked immunosorbent determination of MCP-1 (Monocyte chemotactic protein-1) in blood serum, MCP-1-ELISA-BEST (Vector-Best, Novosibirsk, Russia), according to the manufacturer’s protocol.

### 4.7. Live Cell Imaging

Breast cancer cells were labeled with PKH26 (MINI26-1KT, Sigma-Aldrich Corporation, St. Louis, MO, USA) ac-cording to the manufacturer’s instructions. THP-1 cells were differentiated into macrophages by treatment with phorbol 12-myristate 13-acetate (PMA) at a dose of 10 ng/mL for 24 h. The Hoechst 33342 dye (Lumiprobe, Moscow, Russia) was utilized to label the macrophages at a concentration of 10 µg/mL for a duration of 30 min. The labeled cells were washed with PBS solution and seeded into a 24-well plate, with one well designated for each experimental condition. The concentration was 25,000 cells per well for MCF-7 and MDA-MB-231, and 100,000 cells per well for macrophages. The mono- and co-culturing were conducted in serum-free RPMI medium, both with and without TNF-α (50 ng/mL). Live imaging was conducted using the CellVoyager-CQ1 Benchtop High-Content Analysis System (Yokogawa Electric Corporation, Musashino, Tokyo, Japan), with parameters set at 10X magnification and an image resolution of 2527 × 2135 pixels/1649 × 1393 μM. The following channel set was utilized: Channel 1—“Dapi/Hoechst”—Laser Emission: 405 nm (20%, 16-bit gain “low noise & high well capacity”, exposure: 1750 ms), Excitation Filter: 477/60 nm; Channel 2—“PKH26”—Laser Emission: 561 nm (20%, 16-bit gain “low noise & high well capacity”, exposure: 1000 ms), Excitation Filter: BP617/73 nm; Channel 3—Brightfield, exposure: 250 ms. The experiment lasted 24 h and involved a series of 96 photos taken at 15 min intervals. All photos were obtained from the three fields of view for each well.

### 4.8. Cell Motility Measurement

Cell motility was assessed via the “Manual Tracking” plugin in Fiji distribution of ImageJ software (version 2.14/1.54f; Java.1.8.0_66) [92]. For each experimental condition, three fields of view were analyzed, yielding 50 individual cell tracks per field of view and a total of 150 individual cell tracks per condition. Pixel dimensions were set to 0.6525524/0.6524590 based on the characteristics of the live cell imaging CellVoyager-CQ1 system (image resolution, 2527 × 2135 pixels/1649 × 1393 μm). The comprehensive table of average object velocity measurements is included in Supplementary Tables S1 and S2.

### 4.9. Macrophage Aggregation Measurement

The aggregation of macrophages was evaluated by quantifying object size in the blue channel at the initial (0 h) and final (24 h) time points. Image processing and quantitative analysis were performed using the Fiji distribution of ImageJ software (version 2.14/1.54f; Java.1.8.0_66) [92]. To ensure accurate segmentation of the target structures, a specific background subtraction and filtering strategy was employed. Images were converted to 8-bit grayscale and subjected to the ‘Remove Outliers’ filter (radius = 5 pixels, threshold = 0). This radius was empirically selected to eliminate high-frequency background noise and detector artifacts while fully preserving the morphological integrity of the objects of interest. Following noise reduction, the pre-processed images were binarized using the ‘Convert to Mask’ function. Quantitative assessment was conducted via the ‘Analyze Particles’ tool. A minimum size exclusion threshold of 5 pixels was applied to further prevent the integration of non-specific debris, yielding the total area of the identified particles in square micrometers. Three fields of view were analyzed per experimental group, and the total number of individual aggregates analyzed across these three fields ranged from 1500 to 3000 per group. The entire workflow, including dataset aggregation, was automated using custom ImageJ macros and Python scripts (pandas library). The custom ImageJ macro is provided in Supplementary File S2, and the full data of macrophage aggregate distribution is included in Supplementary Tables S3 and S4.

### 4.10. Transwell Cell Migration Assay

To study the ability of conditioned medium derived from breast cancer cells with different ZEB1 statuses to act as a chemoattractant for macrophages, we used the Transwell migration assay (6.5 mm Transwell^®^ with an 8.0 µm Pore Polycarbonate Membrane Insert, Corning Inc., Corning, NY, USA). The Transwell inserts with a pore diameter of 8 µm were fitted into 24-well culture plates. The upper chambers were filled with 200 µL of serum-free RPMI medium containing 50,000 cells. The lower chambers contained 500 µL of conditioned medium from the breast cancer cells. The conditioned serum-free RPMI medium was derived after 24 h of TNF-α (50 ng/mL) treatment of MCF-7 and MDA-MB-231 cells with different ZEB1 statuses. Both cell lines were seeded at a concentration of 100,000 cells/mL. The control wells contained 10% FBS culture RPMI medium or serum-free medium (with and without the addition of TNF-α) in the lower chambers. Six Transwell inserts were used for each experimental and control medium sample. THP-1 cells were differentiated into macrophages by treatment with phorbol 12-myristate 13-acetate (PMA) at a concentration of 10 ng/mL for 24 h. The macrophages were washed free of FBS-containing complete RPMI medium by centrifugation in sterile PBS (5 min, 700 rpm). After that, the cells were diluted in serum-free RPMI medium and added to the upper Transwell chamber at an appropriate concentration. To prove the role of CCL2 in THP-1 macrophage chemoattraction, we also used the CCR2 receptor inhibitor INCB 3344 (Cat. No. HY-50674, MedChemExpress, Monmouth Junction, NJ, USA) at a concentration of 100 nM. The inhibitor was added to the upper and lower chambers in serum-free and conditioned medium, respectively. After 72 h, the inserts were stained with crystal violet dye for 1 h and washed with water. The upper surface of each insert was gently wiped with a wet cotton swab. After that, all inserts were photographed with a mobile phone camera against a matte white light panel. Slight variations in brightness and contrast between individual photographs are due to the manual acquisition conditions. The relative amount of cell-covered area on the lower surface of chambers was calculated by Fiji distribution of ImageJ software (version 2.14/1.54f; Java.1.8.0_66) [92]. Every image was converted into 16-bit format. Further, using the function “Threshold” in the “Adjust” tab, the 164-point threshold was exposed. The mask of particles below that point on the dark background was acquired. The circle figure with a size corresponding to the area of the Transwell bottom surface was added to the ROI-manager. The same processing parameters were applied to every image, ensuring uniform and comparable quantification across all conditions. Finally, the percentage of area of the circle covered by the mask of particles was calculated (see Supplementary Table S5).

### 4.11. ChIP-Seq Data Visualization

Publicly available ChIP-seq signal tracks (bigWig format) were obtained from the Gene Expression Omnibus (GEO). For MDA-MB-231 cells, the following accession numbers were used: GSM2360622 (ZEB1) [37], GSM2862180 (H3K27me3), GSM2862182 (H3K4me3), and GSM2862183 (H3K27ac) [93]. For MCF-7 cells, ZEB1 ChIP-seq data were obtained under accession GSE190095 [45]. All datasets were aligned to GRCh37/hg19 in the UCSC Genome Browser. The coding region and regulatory elements of the *CCL2* gene were identified using the GeneCards database [94,95] and its integrated GeneHancer regulatory element repository.

### 4.12. Clinical Data Statistical Assay

We utilized breast cancer (BRCA) patient data from the TCGA Pan-Cancer Atlas (PanCanAtlas) project [96]. Annotations for tissue type (tumor/normal), histology (2016 Histology Annotations), and PAM50 molecular subtype were sourced from Supplementary Table S6 of the following publication [97]. Tumor type annotations (tumor type) and RSEM-normalized expression data for the genes ZEB1 and CCL2 were obtained from the cBioPortal website of The Pan-Cancer Atlas (PanCanAtlas) [98]. All variables were consolidated into a unified dataset (Supplementary Table S6). The correlation coefficient between the expression of the ZEB1 and CCL2 genes was assessed using Pearson’s correlation analysis.

### 4.13. Software Used

Statistical data processing and plotting were performed using GraphPad Prism8 Soft-ware. The image processing was conducted in Fiji distribution of ImageJ software (version 2.14/1.54f; Java.1.8.0_66) [92]. Western blot analysis was performed using ImageLab 6.0.1 Software, Bio-Rad. RT-PCR data was analyzed with CFX Manager 3.1 Software, Bio-Rad. Correlation analyses and visualization were performed in Python v3.10.12 using SciPy v1.13.0 for Pearson correlation analysis, NumPy v1.26.4 for linear regression fitting, and Matplotlib v3.8.4 for plotting.

## 5. Conclusions

We have demonstrated that CCL2 expression depends on ZEB1 status in both luminal (MCF-7) and triple-negative (MDA-MB-231) breast cancer cell lines. While ZEB1-positive cells interacted with macrophages in both cell lines, this interaction differentially affected their motility: macrophages facilitated the motility of MCF-7 cells, and they had no effect on MDA-MB-231 cells. The results of our experiments were corroborated by clinical data analysis, which revealed a correlation between ZEB1 and CCL2 expression in Luminal A, infiltrating lobular type, and basal-like breast cancer datasets. Thus, we uncovered the role of ZEB1 in CCL2-mediated cancer—immune cell interactions that likely promote malignancy progression.

## Data Availability

The data presented in this study are available on request from the corresponding author.

## Supplementary Materials

The following supporting information can be downloaded at https://doi.org/10.5281/zenodo.23413806, accessed on 24 September 2026.

## Abbreviations

The following abbreviations are used in this manuscript:

CCL2	C-C motif ligand 2
ChIP-seq	Chromatin immunoprecipitation sequencing
EMT	Epithelial–mesenchymal transition
MET	Mesenchymal to epithelial transition
MCP-1	Macrophage chemoattractant protein 1
TNBC	Triple-negative breast cancer
TNF-α	Tumor necrosis factor α
ZEB1	Zinc finger E-box binding protein
